# Clinical Applications of Mobile Health Wearable–Based Sleep Monitoring: Systematic Review

**DOI:** 10.2196/10733

**Published:** 2020-04-01

**Authors:** Elise Guillodo, Christophe Lemey, Mathieu Simonnet, Michel Walter, Enrique Baca-García, Vincent Masetti, Sorin Moga, Mark Larsen, HUGOPSY Network, Juliette Ropars, Sofian Berrouiguet

**Affiliations:** 1 Urci Mental Health Department Brest France; 2 IMT Atlantique Lab-STICC F-29238 Brest Brest France; 3 EA 7479 SPURRBO Université de Bretagne Occidentale Brest France; 4 Fundacion Jimenez Diaz Madrid Spain; 5 Clinea Psychiatry France Paris France; 6 IMT Atlantique Brest France; 7 Black Dog Institute University of New South Wales Sydney Australia; 8 Please see Acknowledgements for list of collaborators; 9 Laboratoire de Traitement de l'Information Médicale INSERM UMR 1101 Brest France; 10 Department of Child Neurology University Hospital of Brest Brest France

**Keywords:** sleep, eHealth, telemedicine, review, medicine, wearable electronic devices

## Abstract

**Background:**

Sleep disorders are a major public health issue. Nearly 1 in 2 people experience sleep disturbances during their lifetime, with a potential harmful impact on well-being and physical and mental health.

**Objective:**

The aim of this study was to better understand the clinical applications of wearable-based sleep monitoring; therefore, we conducted a review of the literature, including feasibility studies and clinical trials on this topic.

**Methods:**

We searched PubMed, PsycINFO, ScienceDirect, the Cochrane Library, Scopus, and the Web of Science through June 2019. We created the list of keywords based on 2 domains: wearables and sleep. The primary selection criterion was the reporting of clinical trials using wearable devices for sleep recording in adults.

**Results:**

The initial search identified 645 articles; 19 articles meeting the inclusion criteria were included in the final analysis. In all, 4 categories of the selected articles appeared. Of the 19 studies in this review, 58 % (11/19) were comparison studies with the gold standard, 21% (4/19) were feasibility studies, 15% (3/19) were population comparison studies, and 5% (1/19) assessed the impact of sleep disorders in the clinic. The samples were heterogeneous in size, ranging from 1 to 15,839 patients. Our review shows that mobile-health (mHealth) wearable–based sleep monitoring is feasible. However, we identified some major limitations to the reliability of wearable-based monitoring methods compared with polysomnography.

**Conclusions:**

This review showed that wearables provide acceptable sleep monitoring but with poor reliability. However, wearable mHealth devices appear to be promising tools for ecological monitoring.

## Introduction

### Sleep Disorders

Sleep disorders are a major public health issue. Nearly 1 in 2 people experience sleep disturbances during their lifetime [1] with a potential harmful impact on well-being and physical and mental health [2]. The International Classification of Sleep Disorders distinguishes the following 6 categories: insomnia, sleep-related breathing disorders, central hypersomnia, circadian rhythm disorders, parasomnias, and sleep-related motor disorders [3]. For example, insomnia is characterized by complaints about the duration and quality of sleep, difficulty falling asleep, nocturnal awakenings, early awakening, or nonrecuperative sleep [4]. This symptomatology must be present at least three times a week for at least 1 month, with negative consequences the next day. Sleep and mental health are highly related, with many mental health problems also being associated with sleeping disorders [5]. Traditionally, sleeping disorders have been viewed as a consequence of mental health disorders, and evidence also suggests that sleeping disorders can contribute to the development of new mental health problems [6].

### Sleep Monitoring

Normal sleep is characterized by a succession of 4 to 6 cycles lasting approximately 90 min. Each of these cycles consists of slow-wave phases and rapid eye movement (REM) sleep, which are related to the slowdown and activation of the central nervous system. During REM sleep, or stage 5, REMs are observed and muscle tone is abolished. The early-night cycles are especially rich in deep, slow sleep, and the latter cycles are dominated by REM sleep [7]. The duration of normal sleep varies between 6 and 10 hours depending on several factors, the most important of which are age and genetics.

Normal and pathological sleep can be explored either subjectively, that is, by asking the subject, or objectively, using sensors. An epidemiological study conducted in 2013 with over 1000 participants found the prevalence of subjective insomnia to be 15%, whereas the objective prevalence measured by polysomnography (PSG) was 32% [8]. To date, PSG remains the gold standard for objectively assessing sleep characteristics. The polysomnograph plots a hypnogram, integrating data from several sensors: an electroencephalogram (EEG), an electromyogram (EMG), an electrooculogram (EOG), thoracic movement (from belts on the chest and abdomen), airflow measures, oximetry, and an electrocardiogram (ECG). The sleep stages are scored according to standard visual criteria based on the EEG, EOG, and EMG sensors [5]. The assessment must be carried out under controlled conditions in the laboratory for 8 to 12 hours. An automated hypnogram analysis is possible but still requires manual integration of data [7]. Successful recording of the PSG over the course of the recording and the analysis of the results must be carried out by a clinician with expertise in sleep pathologies and brain disorders. Although PSG is considered the *gold standard*, it is an examination with limitations: it can be cumbersome for the patient, is not very accessible, and is not being realized in ecological conditions.

### Mobile Health Wearables

The internet has increased the possibilities for improved patient monitoring. The integration of mobile phones and wearable tools into medical practice has been heralded as the electronic health and mobile health (mHealth) era [9]. These tools can be used to self-monitor or self-assess, allowing individuals to better understand their behavior and body and therefore their health. Aspects of daily life are particularly targeted, with measures of diet, physical activity, or sleep. These self-measurements can be tracked and analyzed with the objective of modifying individual behaviors, including using educational approaches. We therefore observed an association between the concepts of self-monitoring or self-tracking and empowerment, with greater patient involvement and better autonomy. Finally, these devices also allow clinicians to access and review clinical data in real time [10].

### Wearables and Sleep Monitoring

The most frequent sensor embodied into commercially available wearables for sleep monitoring is the actimeter. The actimeter uses an accelerometer worn on the wrist and thus detects the movements of the limb [11]. The use of the actimeter has increased because it is easy to use and allows recordings over periods of time longer than a single night of PSG. However, this assessment method has some limitations. Indeed, according to the numerous comparative studies with PSG, it has been shown that the actimeter hardly detects sleepiness, underestimates the latency of falling asleep, and overestimates the number of microawakenings compared with the reference examination. Finally, this device does not provide information on the stages of sleep. Actimetry is therefore limited to subjects with circadian rhythm disturbances and to evaluation of total sleep time (TST) [12]. Some devices use electroencephalographic and electro-oculographic recordings [13]. However, these devices, still not widely used, require the positioning of several electrodes and are therefore impractical for home use by the patient [12].

Other devices measure heart rate and rely on the variability in the heart rate to identify the stages of sleep. Indeed, this variability is higher during paradoxical sleep or nocturnal awakenings and lower during slow sleep [14] because of the sympathetic or parasympathetic action modulations of the autonomic nervous system [12]. These devices are available in different forms, such as watches, chest bands, electrodes, and monitors on the mattress or pillow, but still have poor results [11].

Overall, wearables are promising sleep-monitoring methods and allow for the recording of several nights, whereas PSG assesses only a single night of recording [11]. A total of 3 reviews have examined the potential features of wearable devices for sleep monitoring [15-17]. However, none of these reviews used a systematic review method to report recent clinical research results. Another review recently assessed the efficiency of actigraphy for evaluating mood disorders [18] and activity [19] but did not have any specific focus on sleep monitoring using mHealth wearable methods. Our hypothesis was that the use of wearables was described in the scientific literature. We therefore conducted a review of the literature on the use of mHealth wearable devices for sleep assessment.

## Methods

### Objectives and Databases

This literature review aims to identify published articles focusing on wearable-based sleep recording in human participants.

We used the Preferred Reporting Items for Systematic Reviews and Meta-Analyses [20] to identify, select, and critically appraise relevant research while minimizing bias.

### Selection Criteria

**Figure 1:**
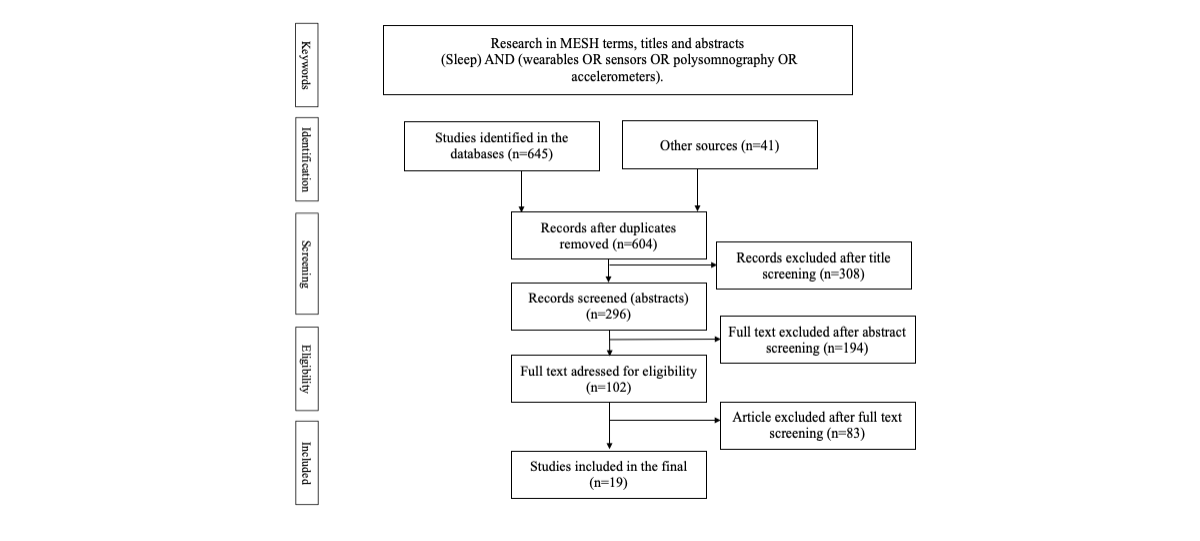
Preferred Reporting Items for Systematic Reviews and Meta-Analyses (PRISMA) flowchart. MESH: Medical Subject Headings.

The literature search was conducted in June 2019 in the PubMed, PsycINFO, Science Direct, Cochrane Library, Scopus, and Web of Science databases. The keywords used were chosen from the terms used in the health terminology of the biomedical reference thesaurus or MeSH terms. The search was conducted using *AND* and *OR* logistic operators in the MeSH terms, titles, and summaries (Figure 1). The keywords and search strategy we used were (sleep) AND (wearables OR sensors OR polysomnography OR actigraphy).

We included randomized controlled trials and nonrandomized studies. We excluded studies without a clinical population, theoretical articles, editorials, and viewpoints without practical results. We excluded articles with an exclusive focus on technical aspects, sleep-monitoring devices that were not connected to the internet, articles presenting monitoring procedures that were not performed ecologically (ie, at home), and unstructured narrative reviews. We also excluded narrative reviews or any article reporting results in an under 18-year-old population.

### Data Extraction and Analysis

The analysis of the articles was conducted in two stages. As a first step, a census based on the review of titles and abstracts of scientific papers meeting the inclusion criteria was conducted. Two review authors (SB and EG) independently assessed all studies retrieved against the inclusion criteria. Disagreements were resolved by a third review author (JR). During the literature review process, relevant studies were categorized using a 2-step approach. We first performed a review of the titles and abstracts of all publications that were identified as relevant according to the inclusion criteria. Abstracts were then categorized by the type of methodology used, health condition, applications, and purposes. The full texts of all publications that were not excluded during the title and abstract review stage were checked. Publications that met all inclusion criteria comprised the final sample. The full texts of all publications that were not excluded after the analysis of titles and abstracts were reviewed. All studies meeting the inclusion criteria were included.

## Results

### Identification of the Articles

The steps of the literature review research and analysis are summarized in Figure 1. The initial search identified 645 articles. After the removal of duplicate articles, screening based on the titles resulted in the removal of 308 articles. A total of 194 articles were excluded after review of the abstracts. After review of the full text, 83 additional articles were excluded because they did not meet the inclusion criteria. Thus, 19 articles meeting the inclusion criteria were included in the final analysis—an overview of these studies is shown in Multimedia Appendix 1.

### Design and Size

Of the 19 studies in this review, 58% (11/19) were comparison studies with the gold standard, 21% (4/19) were feasibility studies, 16% (3/19) were population comparison studies, and 5% (1/19) assessed the impact of sleep disorders in the clinic. The samples were heterogeneous in size, ranging from 1 to 15,839 patients.

### Analysis of Results

A review of the full text of the articles revealed 4 categories. We identified feasibility studies, population comparison studies, studies comparing mHealth wearables to PSG to identify sleep stages, and a study describing the impact of sleep on clinical outcomes.

#### Feasibility Studies

In their study, Baron et al [21] aimed to outline the theoretical foundation and iterative process of designing the *Sleep Bunny*, a technology-assisted sleep extension intervention including a mobile phone app, a wearable sleep tracker, and brief telephone coaching. The population comprised 6 adults with short sleep duration (<7 hours), testing the application with the sleep tracker, and the telephone coaching once a week over 4 weeks. The survey, based on open-ended questions, asked participants to provide comments for general feedback on the content and layout of the app. In conclusion, users enjoyed the wearable sleep tracker and found the app visually pleasing but suggested improvements to the notification and reminder features.

The team of Castiglioni et al [22] studied the feasibility of wearing a *MagIC-SCG* sternal wrist device during high-altitude sleep, which is conducive to hypoxia. Their device recorded an ECG, respiratory movements, sternal accelerations, and oxygen saturation. The study demonstrated the feasibility of recording and using the equipment in high-altitude conditions.

The study by Di Rienzo et al [23] demonstrated the feasibility of estimating cardiac functions such as contraction and isovolumic relaxation times or ventricular ejection time during sleep. The data were transmitted in real time to an external device via a Bluetooth connection.

Kayyali et al [24], in the United States, investigated the feasibility of a wearable sleep recorder at the patient’s home. Their device, *PSG @ home*, was placed in the thoracic region and recorded respiratory movements, oxygen saturation, airflow, snoring, body position, and an ECG. Their study demonstrated the feasibility of using this discrete device overnight at the subject’s home.

#### Population Comparison

The purpose of the study by Fagherazzi et al [25] was to highlight the determinants involved in poor sleep. The authors calculated the 7-consecutive-night deep sleep/total sleep ratio of a large number of users of Withings wearable devices. They used an algorithm that used the data obtained from both the accelerometer and temperature sensor. A ratio indicating poor sleep was defined as below 0.40. Their findings showed that young men with elevated heart rate and high blood pressure were at higher risk for poor sleep quality.

Migliorini et al [26] compared sleep records between a healthy adult population and a patient with bipolar disorder. The monitoring was performed by the *Smartex* T-shirt equipped with sensors. The data collected were an ECG, respiratory activity. and movement via an accelerometer, allowing the stages of the sleep and an estimation of the percentage of paradoxical sleep to be obtained. The results showed a variability in the reduced heart rate in the individual with bipolar disorder, as well as an increase in the percentage of paradoxical sleep. These results need to be confirmed by a larger sample but seem to be an interesting way of identifying emergent depressive disorders.

The study by Sringean et al [27] compared the sleep of individuals with Parkinson disease in the homes with that of their spouses or partners as healthy controls to provide a quantitative analysis of nocturnal hypokinesia. Wearable sensors were worn on the trunk and limbs. Records included number, speed, acceleration, degree, and duration of movement/turnarounds, number of bed exits, and limb movements. The researchers noted the effectiveness of their system to record nocturnal movements, demonstrating the significant presence of nocturnal hypokinesia.

#### Comparison With Polysomnography

Comparisons with PSG were conducted using either commercially available devices or custom wearable devices developed specifically for the study.

#### Commercially Available Devices

The American team of De Zambotti et al [28] compared data from the Jawbone UP with PSG data collected simultaneously. The Jawbone UP is a wristband that, in its first version, records accelerometer data. Comparisons were made between TST, bedtime, sleep latency, and nighttime awakenings. It has been shown that the estimates of these parameters are in good agreement with PSG, a reference examination for sleep pathologies.

Kang et al [20] compared the commercial Fitbit Flex device with PSG in terms of the accuracy of detecting sleep epochs. They studied a population of 41 individuals with insomnia and 21 good sleepers. Participants wore the wearable electronic device while undergoing PSG for 1 night. The measures of interest in this study were TST, sleep efficiency (SE), sleep onset length, and wake after sleep onset (WASO). They concluded that the frequency of agreement was high in good sleepers but significantly low in those with insomnia.

In their pilot study, Looney et al [29] compared electroencephalographic recordings obtained with standard electrodes at the level of the scalp and those obtained with an intra-auricular device simultaneously during sleep. The lines were read blindly by an expert. The results showed a significant concordance between the two recordings.

Parak et al [30] compared the nightly heart rate recording of the connected watch *PulseOn* with the reference test, the ECG. The study, conducted at home, showed that the device correctly detected 99.57% of heartbeats, making it an accurate method during sleep.

Mantua et al [31] compared the data from 5 portable connected devices recording sleep with those of the gold standard, PSG. The devices studied were Actiwatch, Basis, Misfit Shine, Fitbit Flex, and Withings Pulse O2. The recordings were made simultaneously at the participant's home, with participants wearing the 5 devices on the wrists, and PSG was performed. Significant data loss was reported by *Fitbit Flex* and *Misfit Shine*. The correlation analysis allowed them to conclude that there was no significant difference in estimating TST between PSG and each of the 5 devices. In addition, only *Actiwatch* had concordant data with the baseline SE test. The light sleep time differed between all devices. Finally, a correlation of deep sleep time was significant only for *Basis*.

Liang et al [32] aimed to examine the accuracy of Fitbit Charge 2 for measuring transition probabilities among wake, light sleep, deep sleep, and REM sleep under free-living conditions. A Fitbit Charge 2 and a medical device were used concurrently to measure a whole night’s sleep in participants’ homes. Sleep data were collected from 23 participants.

Fitbit had the tendency to overestimate the probability of staying in a sleep stage while underestimating the probability of transiting to another stage. SE>90% (*P*=.05) was associated with a significant increase in measurement error. A Pittsburgh Sleep Quality Index (PSQI)<5 and WASO<30 min could be associated with significantly decreased or increased errors, depending on the outcome sleep metrics.

Dafna et al [33] studied the use of a wearable respiratory sound–recording tool, with the aim of estimating respiratory rate by analyzing the audio signal. The data were compared with those of PSG. The authors concluded that their method was reliable and robust for estimating the respiratory rate. The device was described to be not intrusive and did not interfere with the subject’s sleep.

Sano et al [34] compared EEG data with the Q Sensor Affectiva for the detection of waking and sleeping phases in a specialized hospital laboratory. Q Sensor Affectiva is a watch that records skin temperature, cutaneous conductance, and acceleration. In their conclusion, it appears that the combination of acceleration and skin temperature is most effective for the sleep/wake classification.

Sargent et al [35] evaluated the validity of a commercial wearable device, the Fitbit HR Charge, for measuring TST. This study showed that the Fitbit HR Charge overestimated TST for night-time sleep periods and for daytime naps.

#### Custom Devices

The team of Kuo et al [36] developed and evaluated a hand-held wrist-based sleep-recording tool based on actimetry. The wearable device was judged to be energy efficient and highly accurate in measuring SE, TST, sleep time, and nighttime awakenings. PSG measurements were taken simultaneously. The different variables were concordant and significantly correlated with TST and SE. According to the authors, this system is an interesting option for obtaining objective sleep data at the patient’s home.

Rodriguez-Villegas et al [13] compared the effectiveness of a wireless system for the detection of apnea and hypopnea with that of PSG. The 17-gram device was placed on the skin of the anterior aspect of the neck. It recorded turbulence in the trachea using an acoustic chamber. Data were analyzed by blinded investigators. The tolerance of the device was greater than that of PSG. However, the results did not agree with the gold standard regarding the correct detection of hypopneas. In conclusion, this tool could be an adequate solution for the monitoring of apneas in ecological conditions but would not replace a complete recording in the sleep laboratory.

#### Impact of Sleep on Clinical Outcomes

In the study by Agmon et al [37], the impact of sleep on walking performance in institutionalized elderly individuals was measured using a connected watch and an accelerometer. SE, sleep latency, TST, and nocturnal awakenings were taken into consideration. The team demonstrated that a decrease in recovery sleep was significantly associated with a decrease in start-up speed and a greater variability in walking during double tasks.

## Discussion

### Principal Findings

This review of the literature shows an increasing interest in the use of wearable devices for sleep assessment. Overall, our review shows that mHealth wearable–based sleep monitoring is feasible but not reliable. Existing commercial technology might be attractive for both clinicians and patients, as shown by the excellent acceptance of mHealth wearable technologies we found. This acceptance has clearly influenced the feasibility of ecological sleep monitoring methods in the selected studies. However, we identified some major limitations to the reliability of wearable-based monitoring methods compared with PSG.

### A Global Lack of Reliability

Our study and recent findings indicate that wearables are reliable monitoring tools compared with PSG. However, some recent findings have shown that these devices often over- or underestimate TST or total wake time. This lack of reliability might be partially explained by the power of the trials. For example, only 7 studies among 18 included 30 participants or more [38,39]. Furthermore, recent findings also emphasize that little is still known about physiological monitoring in ecological situations [40], which might explain some discrepancies in results obtained ecologically compared with the gold standard in sleep-recording laboratories. Furthermore, the most common recording methodology identified in this review was motion sensing via accelerometry, in which, recording limits are well established [16]. The research literature consistently shows that wrist accelerometry, even in healthy adults, has high sensitivity but low specificity for sleep detection. The study by Liang [32] showed that Fitbit Charge 2 underestimated sleep stage transition dynamics compared with the medical device. Fitbit had the tendency to overestimate the probability of staying in a sleep stage while underestimating the probability of transiting to another stage. SE>90% (*P*=.047) was associated with a significant increase in measurement error. PSQI<5 and WASO<30 min could be associated with significantly decreased or increased errors, depending on the outcome sleep metrics. A significant improvement in ecological sleep parameter detection might be provided by recent improvements in miniaturized sensors [41] and embodied data analysis methods.

### Limitations of the Review Method

Although the studies selected for this review are recent, the rapid evolution of technologies in this area makes it difficult to adjust research to keep pace with commercial releases. Since the start of the review process, additional articles may have been published. Moreover, while the conclusions are encouraging, most of them are pilot studies with small samples. Limits on scientific validity mean that these devices are not usable in the current clinical setting, and it may be premature to recommend them. Given the rapid progression of technologies, it does not seem unrealistic to think that more complete and validated devices will be available soon. Moreover, the heterogeneity of the population studied in this review makes it difficult to draw a general conclusion. Our review reflects the broad spectrum of usability of mHealth wearable devices in the field of sleep. Finally, it should be noted that this review of the literature does not provide any data on the use of these objects in the long term because the studies included were mostly short-term clinical trials [29] with devices that may have defects in the collection of data because of limited battery life, for example.

Data mining is the core of analysis and is used to explore clinical questions in large databases as those produced by mHealth wearables. The data mining process includes several steps, including data selection, data processing, and machine learning, to identify which factors may influence results. This review did not identify any studies describing the data mining techniques employed. This finding might be explained by the editorial policies of the clinically oriented articles our inclusion criteria selected. Another reason is that mHealth wearables are commercial products, and the analysis methods are patented. As an example, Fitbit devices that were used in several studies do not give access to the raw data gathered by Fitbit wearables, which make these devices hardly suitable for medical purposes. Consumer sleep devices contribute to the blurry boundary between sleep as a medical concept and sleep as *wellness* and the need for a framework to interpret consumer sleep device outputs.

### Future Applications and Recommendations

Regarding the results of our review and as a proposal for future applications and development, some recommendations can be made.

#### Make Sleep Data Readily and Remotely Available for Nurses and Physicians

Our review shows that sleep can be monitored using wearables for an extensive panel of physical and mental conditions. Most of the research used commercially available devices that were linked to a mobile phone, increasing the networking capabilities and the user experience [28-30]. Collected data can be processed and transferred over the internet to a remote clinical back-end server for further analysis, assessment, decision making, and intervention. However, we noted that the potential to explore sleep remotely and in real time has been poorly reported. Recent research has specifically focused on comparing the reliability of wearables to monitor sleep with PSG. The ability to capture that data, apply machine learning to evolving trends, and alert patients, nurses, and physicians instantaneously is powerful. As sleep is a risk factor for many chronic diseases, the momentary tracking of everyday sleep quality of patients may be very useful for a wide range of clinical conditions, including mental health disorders, neurological disorders, and other chronic diseases. Thus, innovative procedures aiming to make (even simpler than PSG-like signals) outpatient sleep records accessible to clinicians are needed.

#### The Future of Wearable Sleep Monitoring: Long-Term Assessment

Sleep quality is a key component of health and well-being. Our review shows that most wearables lack the ability to monitor sleep with the same accuracy as PSG. Another important limitation to note is that sleep monitoring using wearables has been poorly explored in a long-term setting. The study duration did not exceed 1 month. However, the main advantage of wearable PSG is that the recording of sleep can be performed over a long period. This ability might help to strengthen or reveal the links between sleep quality and health outcomes, such as depression [42], respiratory problems [43], and epilepsy [44]. Thus, we recommend long-term sleep-monitoring studies.

#### Development of Specific Sleep-Monitoring Devices

Acceptance is one of the key components of implementation in the clinical setting of wearables. A recent review showed that designing an all-purpose wearable activity tracker (WAT) is unreasonable [19]. A variety of design concepts and data models should continue to emerge that align with the personal preferences of various groups of users. However, it is important to note that most commercially available wearables described in our review have been developed for activity tracking. Sleep monitoring is often presented as a secondary feature of activity trackers. Although some specific sleep-monitoring devices exist, the further development and assessment of devices aiming to specifically monitor sleep are needed. Furthermore, these devices should take more advantage of existing mHealth features, as goal-based gamification, continuous feedback, and social support seem to encourage healthy sleep behaviors.

#### Commercially Available Versus Custom Wearable Activity Trackers

Our reviews show that most research focuses on commercially available WATs. This important limitation reflects the lack of cooperation among device industries, information technology scientists, and clinical researchers, who might be tempted to implement commercially available wearables instead of developing expensive customized hardware devices. A major limitation of commercially available devices is the poor accessibility of data for analysis purposes, especially in the clinical population. However, analyzing the data generated by commercial wearables is feasible. These data sets are orders of magnitude larger than traditional research studies and can be accessed by researchers at a relatively low cost [45]. However, the consumer market of wellness claims is not necessarily adapted to clinical practice settings and, as a result, may reduce the adoption of these devices in clinical practice by both patients and clinicians. Overall, we believe that further studies should incorporate device developments to better fit long-term and reliable ecological sleep monitoring.

### Conclusions

This review of the literature on mHealth wearable devices for sleep monitoring shows the growing interest in these new technologies, as well as their wide application. Indeed, it was observed that the studies can reflect different specialties of medicine and that the populations studied varied. In addition, this interest is recent, with the majority of studies from 2014 or after. Qualitatively, the majority of devices were considered comfortable [30], easy to use [24], and to preserve the natural sleep of the user [29,33], making them good candidates for home monitoring and care [13,26,36]. In addition, the wearable devices have an economic advantage, and the preliminary results of this study show a good correlation with the reference examination [28]. Given the many benefits, we must consider mHealth wearable devices as promising tools for ecological sleep monitoring. Our review also highlighted some limitations that may help clinicians and researchers better identify current challenges in ecological sleep monitoring using wearables.

## Appendix

Multimedia Appendix 1 Summary of the selected studies sorted by year.

## Abbreviations

ECG: electrocardiogram
EEG: electroencephalogram
EMG: electromyogram
EOG: electrooculogram
mHealth: mobile health
PSG: polysomnography
PSQI: Pittsburgh Sleep Quality Index
REM: rapid eye movement
SE: sleep efficiency
TST: total sleep time
WASO: wake after sleep onset
WAT: wearable activity tracker
