# Supplementary material for: Clinical Applications of Mobile Health Wearable–Based Sleep Monitoring: Systematic Review
Source: JMIR Mhealth Uhealth. 2020 Apr 1;8(4):e10733. doi: 10.2196/10733 (PMC7160700; doi:10.2196/10733)
Supplement: Multimedia Appendix 1 [file mhealth_v8i4e10733_app1.docx]

**Multimedia appendix 1.** Summary of the selected studies sorted by year.

| Authors, year, country | Population (n) | Inclusion criteria | Exclusion criteria | Aim | Method | Outcomes | Number of nights | Results |
| --- | --- | --- | --- | --- | --- | --- | --- | --- |
| Baron et al, 2018, the United States [20] | Participants with short sleep duration (6.) | 18-65 years and self-reported sleep duration <7 hours per night. | Unstable or serious medical conditions, shift work, obstructive sleep apnea or high risk for apnea, other sleep disorders, current series of unstable psychiatric disorders, and pregnancy or desire to become pregnant. | To outline the theoretical foundation and iterative process of designing the *Sleep Bunny*, a technology-assisted sleep extension intervention including a mobile phone app, wearable sleep tracker, and brief telephone coaching. | Mobile phone app, wearable device, and telephone coaching once a week for 4 weeks. | Open-ended surveys. | 28 | Users enjoyed the wearable sleep tracker and found the app visually pleasing, but suggested improvements to the notification and reminder features. |
| Kayyali et al, 2008, the United States | Adult patients with fibromyalgia (10). | Volunteers. | None. | To assess the feasibility of home monitoring of sleep disorders using small wireless technologies. | A 14-channel thoracic wearable wireless monitor, including video, streaming in real time from the patient’s home. | Chest and abdominal respiration effort, pulse oximetry, airflow, snoring, body position, and ECG^a.^ | 1 | All 10 studies were successful and generated high-fidelity recordings. |
| Migliorini et al, 2011, Italy | Bipolar adult patients and healthy subjects (9.) | 18-45 years. | Psychiatric disorders. | To compare HRV^b^ during sleep in 2 populations. | Evaluation during sleep in a group of healthy subjects and one with bipolar disorder. | HRV signal and activity signals, ECG. | 1 and 4 | Reduced HRV, increased REM^c^ sleep. |
| Castiglioni et al, 2012, Italy | Male healthy subjects participating in a scientific expedition on Monte Rosa (6). | Volunteers. | None. | To evaluate whether SCG^d^ can detect cardiorespiratory alterations during sleep at high altitude. | Sternal SCG, « MagIC-SCG », at high altitude and sea level for 2 of the participants. | ECG with R-peak, respiratory movements, sternal accelerations, and oxygen saturations. | 1 or 2 | Feasibility demonstrated. |
| Di Rienzo et al, 2014, Italy | Healthy subject (1). | Volunteer. | None. | Feasibility of a beat-to-beat estimation of cardiac mechanical features from the joint ECG and SCG assessment. | ECG, respiration, and SCG, recorded with a sensorized vest with an accelerometer through a Bluetooth connection. | RR interval, pre-ejection period, isovolumic contraction time, left ventricular ejection time, and isovolumic relaxation time. | 8 hours recorded, two 30-min sleep data segments studied. | The beat-to-beat estimation could be obtained. |
| Rodriguez-Villegas et al, 2014, the United Kingdom | Adult patients with sleep apnea and healthy controls (30). | Likely to have spontaneous apnea events. | None. | To create and clinically test a novel miniature medical device, and to test its ability to detect individual events during controlled conditions and during spontaneous sleep. | The SOMNO clinical system was fixed to the skin on the neck in a sleep study room. Data were blindly analyzed. | Turbulence in trachea detected with an acoustic chamber. Finger oximetry, oronasal airflow sensors, thoracic and abdominal expansion bands, and ECG. | 1 | It can be a real solution for apnea home monitoring. The tolerability was superior to the wearable PSG^e^. |
| Sano et al, 2014, the United States | College students (15). | Volunteers. | None. | To present the comparison of sleep-wake classification using EEG^f^ and multimodal data from a wrist-wearable sensor. | In a hospital sleep laboratory; participants wore electrodes for EOG^g^, EEG, and EMG^h^ on their chin and a wrist sensor (Q Sensor by Affectiva) on their dominant hand. | EEG, skin conductance, ST^i^, and acceleration ACC^j^ | 1 | EEG features showed 83% while features from a wrist-wearable sensor showed 74%, and the combination of ACC and ST played more important roles in sleep/wake classification. |
| Dafna et al, 2015, Israel | Adult volunteers (204). | Volunteers. | None. | To compare audio-based breathing rate estimation and the gold standard of PSG. | Use of a digital audio recording device connected to a noncontact microphone that was placed 1.0 m above the patient's head | Breathing sounds | 1 | Reliable and robust method for the estimation of breathing rate. |
| De Zambotti et al, 2015, the United States | Midlife adult women (28) | Volunteers | None | To assess the validity of Jawbone UP compared with that of PSG. | PSG and Jawbone UP data were simultaneously collected. | Total sleep time, time in bed, sleep onset latency, and wake after sleep onset. | 1 | Good agreement in the overall estimation of sleep. |
| Parak et al, 2015, Finland | Healthy adult volunteers (10). | Volunteers. | None. | To evaluate the accuracy of the beat-to-beat detection of the PulseOn consumer wearable optical heart rate monitor. | The comparison was performed against the Firstbeat Bodyguard 2 wearable RR interval recorder which was used as an ECG-based reference. | Beat-to-beat heart rate and the reference RR intervals. | 1 | PulseOn correctly detected 99.57% of the heart beats. It provides an accurate method for long-term HRV monitoring during sleep. |
| Kuo et al, 2016, Taiwan | Male and female, 20-60 years (81). | 56 with good sleep efficiency; 25 with poor sleep efficiency. | None. | To develop a hardware and software integration system that had good performance for wake-sleep staging and assessment of sleep measurements. | Simultaneous PSG measurements, concordance of various sleep measurements between the manual PSG scoring and the wristwatch actigraphy recorder. | EEG, EOG, EMG; actigraphy (accelerometer signals, the peak-to-peak interval, and maximum magnitude are used); SE^k^, TST^l^, SOT, WASO^m^. | 1 (8 hours) | Robustness and reliability of the wearable actigraphy system for the in-home screening of objective sleep measurements (SE and ST). |
| Looney et al, 2016, the United Kingdom | Healthy men, aged 25-36 years (4). | Volunteers. | History of snoring, sleep disorders, or neurological disease. | A pilot study, to determine the agreement between the ear-EEG sensor and gold standard EEG. | EEG simultaneously from the in-ear and standard on-scalp electrodes; a clinical expert, blinded. | EEG. | Naps, 45 min; 360 epochs | Substantial agreement in the detection of N2/N3 sleep with the sleep monitor. |
| Mantua et al, 2016, the United States | Bipolar adult patient and healthy subjects (9). | 18-30 years. | History of sleep or neurological disorders. | To determine the validity of 5 wearable devices: Basis Health Tracker, Misfit Shine, Fitbit Flex, Withings Pulse O2, and a research-based actigraph, Actiwatch Spectrum. | In the participants’ home. They completed a sleep diary. The Wilcoxon Signed Rank tests were used to assess differences and correlational analysis to assess the strength of the relationship. | TST, SE, light sleep time, deep sleep time (from the 4 commercially available monitoring devices); TST, SE (from the research-validated actigraph). | 1 | Strong correlation of TST. SE did not differ from PSG for Actiwatch. Light sleep time differed. Measures of deep sleep time did not differ from PSG (SWS + REM) for Basis. |
| Agmon et al, 2016, Israel | Community-dwelling older adults (34). | 60 or older, can speak, understand, and read Hebrew, independent in activities of daily living including walking. | Presence of neurological diagnoses, severe orthopedic restriction, and significant hearing or vision loss. | To assess the relationship between sleep behavior and gait performance under single-task and dual-task walking conditions. | Wrist activity monitors sleep, logs were used to determine sleep onset and sleep offset; activity data were downloaded and analyzed using ActionW software. | SE, sleep latency, sleep duration, and WASO. | 5 | Lower SE is associated with decreased gait speed and increased gait variability under dual-task conditions. |
| Sringean et al, 2016, Thailand | Parkinson disease (PD) couples (38). | Patients with a diagnosis of PD who had spouses whose age did not differ from theirs by more than 10 years. | Patients who were bedridden, a history of other neurological muscle and joint diseases, a history of sedative drug use. | To quantitatively compare nocturnal movements of PD patients with those of their spouses and to correlate these parameters with disease severity scores. | Wearable sensors (wrists, ankles, and trunk) at the patients’ homes. Sleep diary, and video recording. | Number, velocity, acceleration, degree, and duration of rolling over, number of times getting out of bed, and limb movements. | 1 | It demonstrated the effectiveness for capturing nocturnal movements in patients with PD compared with their spouses. |
| Fagherazzi et al, 2017, France | Adult customers (15,839). | Customers having purchased and used at least 3 connected devices from the consumer electronics company Withings. | None. | To identify the determinants of poor sleep. | Ratio of deep/total sleep as a proxy of sleep quality (in association with available data on age, sex, weight, heart rate, steps, and diastolic and systolic blood pressures). | Total and deep sleep durations. | 7 | Those at risk of having a poor ratio were more frequently male and younger and had an elevated heart rate and high systolic blood pressure. A direct association with weight was observed for total sleep duration exclusively. |
| Kang et al, 2017, Korea | Adult insomnia disorder patients and good sleepers in Gil Medical Center (62). | 18-60 years, history of illness lasting at least 3 months, diagnosed with insomnia disorder based on the DSM-5, Pittsburgh Sleep Quality Index score of ≥8 (or <4 for good sleepers), an apnea-hypopnea index of <15 in PSG. | Hypnotics or psychotropic medication or having been treated with CBT-I during the previous 2 weeks, shift workers or travelers experiencing frequent jet lag, periodic limb movement index during sleep in PSG, other major psychiatric disorders. | To compare the accuracy of the commercial Fitbit Flex device with PSG for detecting sleep epochs. | Participants wore a Fitbit Flex device and actigraph while undergoing overnight PSG. | EEG, EMG, EOG, nasal pressure, thoracic and abdominal effort, oximetry, body position; TST, SE, sleep onset length, WASO. | 1 | The correlation of TST was excellent in both groups, and the frequency of agreement was high in good sleepers but significantly lower in patients with insomnia. |
| Sargent C et al, 2018, Australia | Well-trained adult young athletes (12). | Semiprofessional soccer players, training 5 times per week, playing 1 game on the weekend. Field-based training sessions 3 times a week, gym-based training sessions twice per week. | Clinical diagnosis of a sleep disorder. | To evaluate the validity of a commercially available wearable device for measuring total sleep time. | Participants wore a Fitbit HR Charge on their nondominant wrist and had electrodes attached to their face and scalp to enable polysomnographic recordings of sleep in the laboratory. | TST. | 30, 20 naps | Compared with PSG, the Fitbit overestimated TST for nighttime sleep periods and for daytime naps. |
| Liang et al 2019, Japan | Healthy adult participants (24). | Adult volunteers. | None. | To examine the accuracy of the Fitbit Charge 2 in measuring sleep stages under free-living conditions. | Comparing Fitbit and polysomnograph data. | Transition probabilities among wake, light sleep, deep sleep, and REM sleep under free-living conditions. | 3 | Fitbit Charge 2 underestimated sleep stage transition dynamics compared with the medical device. |

^a^ECG: electrocardiogram.

^b^HRV: heart rate variability.

^c^REM: rapid eye movement.

^d^SCG: seismocardiography.

^e^PSG: polysomnograph.

^f^EEG: electroencephalogram.

^g^EOG: electro-oculogram.

^h^EMG: electromyogram.

^i^ST: skin temperature.

^j^ACC: .

^k^SE: sleep efficiency.

^l^TST: total sleep time.

^m^WASO: wake after sleep onset.
